# Living With School‐Aged Children and the Risk of Absenteeism Among Healthcare Workers During the Twindemic of COVID‐19 and Influenza

**DOI:** 10.1111/irv.70100

**Published:** 2025-04-21

**Authors:** Shohei Yamamoto, Tetsuya Mizoue, Maki Konishi, Kumi Horii, Wataru Sugiura, Norio Ohmagari

**Affiliations:** ^1^ Department of Epidemiology and Prevention Center for Clinical Sciences, Japan Institute for Health Security Tokyo Japan; ^2^ Infection Control Office National Center for Global Health and Medicine, Japan Institute for Health Security Tokyo Japan; ^3^ Center for Clinical Sciences Japan Institute for Health Security Tokyo Japan; ^4^ Disease Control and Prevention Center Japan Institute for Health Security Tokyo Japan

**Keywords:** absenteeism, children, COVID‐19, healthcare worker, influenza, Twindemic

## Abstract

**Background:**

The impact of the simultaneous circulation of COVID‐19 and seasonal influenza, termed the “Twindemic,” on absenteeism among healthcare workers (HCWs), particularly those with children, remains unclear. We aimed to investigate the associations of living with school‐aged children with the risk of SARS‐CoV‐2 and influenza infections and the risk of absenteeism due to own‐ or family‐related COVID‐19 or influenza events among HCWs during the Twindemic.

**Methods:**

This prospective study followed 1473 HCWs at a tertiary hospital in Tokyo from December 2023 to March 2024. We tracked the incidence of SARS‐CoV‐2 and influenza infections and absenteeism due to COVID‐19 or influenza‐related family events. We estimated the hazard ratios (HRs) or incidence rate ratios (IRRs) of these outcomes across living arrangements, focusing on cohabitation with school‐aged children.

**Findings:**

HCWs living with younger school‐aged children (≤ third grade of junior school) had a higher risk of SARS‐CoV‐2 and influenza infections, with HRs [95% confidence interval (CI)] of 1.90 [0.97–3.69] and 2.42 [1.04–5.66], respectively, compared with those living with cohabitants without school‐aged children. Additionally, they showed a higher IRR [95% CI] for absenteeism due to family‐related events (10.9 [4.88–24.5]), and their overall IRR [95% CI] of absenteeism due to own/family‐related events was 3.76 [2.59–5.46].

**Conclusion:**

The Twindemic has heightened absenteeism risks among HCWs with school‐aged children, emphasizing the need for targeted support to help HCWs manage both their professional duties and family responsibilities during such challenging times.

## 1 Introduction

As the COVID‐19 pandemic prolonged and the countermeasures against COVID‐19 became relaxed worldwide, the circulation of seasonal influenza has resumed at pre‐COVID‐19 pandemic levels [1], resulting in the co‐circulation of COVID‐19 and influenza, referred to as “Twindemic.” This situation can further compound burdens on healthcare workers (HCWs) and healthcare systems, which are already under strain due to COVID‐19 [2, 3].

Absenteeism (i.e., leave from work) in relation to SARS‐CoV‐2 or influenza infection among HCWs is a critical issue affecting healthcare systems [4, 5, 6]. Given that household transmission from children is one of the main routes of these infections [7, 8], HCWs living with children might have higher risks of absenteeism due to these infections than those living without children. In addition, HCWs living with children could have a high risk of absenteeism for their responsibility of caring for infected children. Previously, we reported that HCWs living with school‐aged children had a 2‐fold higher risk of absenteeism due to SARS‐CoV‐2 infection or quarantine due to SARS‐CoV‐2 infection of family members than those without school‐aged children during the winter of 2021–2022 season (December to March) in Japan, when only COVID‐19 was epidemic [9].

So far, the impact of the Twindemic on absenteeism among HCWs with children is unclear. During the winter of 2023–2024 in Japan, a significant outbreak of COVID‐19 and influenza caused a number of these infections among children and widespread school closers [10]. This situation prompted us to investigate the association between living with children and the risk of absenteeism due to own infection or family care for COVID‐19 or Influenza‐related events among the staff of a tertiary referral hospital in Tokyo.

## 2 Methods

### 2.1 Study Setting

In the National Center for Global Health and Medicine (NCGM) in Tokyo, Japan, a repeat serological study was launched in July 2020 to monitor the spread of SARS‐CoV‐2 infection among staff during the COVID‐19 epidemic. The details of this study have been reported elsewhere [11, 12]. In the survey, we collected information on COVID‐19‐related factors, including vaccination, occupational infection risk, and infection prevention practices via a questionnaire. From the surveys in 2023, we additionally collected influenza‐related information on vaccination and infection histories to investigate the impact of influenza during the Twindemic. Written informed consent was obtained from all the participants. This study was approved by the NCGM Ethics Committee (approval number:NCGM‐G‐003598).

In the present study, we analyzed participants who attended the ninth survey conducted in December 2023 and its follow‐up survey in March 2024. Of 1656 participants in the ninth survey, data for 1473 who attended the follow‐up survey were used for the present study.

### 2.2 Ascertainment of SARS‐CoV‐2 and Influenza Infection

To assess the influence of co‐circulation of COVID‐19 and influenza between December 2023 and March 2024 (Figure S1), we followed the participants for the incidences of SARS‐CoV‐2 and influenza infections using the in‐house registry administrated by the NCGM Hospital Infection Prevention and Control Unit, which provided information on the date of diagnosis, diagnostic procedures, symptoms, and hospitalization. As per the NCGM rule, staff should undergo diagnostic tests (PCR or antigen tests) for influenza and COVID‐19 when they have cold‐like symptoms, and if the test results are positive, they must report the results to the NCGM Hospital Infection Prevention and Control Unit. In addition, we asked participants if they had contracted COVID‐19 or influenza since the baseline via the questionnaire at the follow‐up survey.

### 2.3 Absenteeism due to Family Care

We asked participants about the number of cohabitants; then, among those with cohabitants, we further asked whether they took a leave due to family care in relation to COVID‐19 or influenza events between December 2023 and March 2024. For those who experienced absenteeism due to family care, we asked them to select the details of their absenteeism from the following choices with multiple responses permitted: children's infection with COVID‐19, children's infection with influenza, school closure due to COVID‐19, school closure due to influenza, non‐child family's infection with COVID‐19 or influenza, and others.

### 2.4 Living Arrangement

We asked participants about their living arrangements via the questionnaire in the follow‐up survey. Participants were categorized into four groups: living alone, living with family, not including school‐aged children, living with younger school‐aged children, and living with older school‐aged children. Young school‐aged children were defined as children in nurseries, in kindergartens, in the first to third grades of elementary school, and with disabilities, and older school‐aged children were children in the fourth to sixth grades of elementary school, in junior and high schools, and university students. If the participants lived with younger and older school‐aged children, they were categorized into the younger group.

### 2.5 Statistical Analysis

We established four outcomes: (1) SARS‐CoV‐2 infection, (2) influenza infection, (3) absenteeism due to family‐related SARS‐CoV‐2 or influenza events, and (4) absenteeism due to own/family‐related events (i.e., a cumulative count of absenteeism due to SARS‐CoV‐2 infection, influenza infection, and family care).

We calculated the person‐time from the date of the baseline survey (December 4–18, 2023) to the date of infection, receiving an additional vaccine, or censoring at the follow‐up survey (March 5–15, 2024), whichever occurred first. We used a Cox proportional hazards model to estimate the hazard ratio (HR) of SARS‐CoV‐2 and influenza infections across the living arrangements. We used a robust Poisson regression model to estimate the incident rate ratio (IRR) of absenteeism due to family‐related events and own/family‐related events across the living arrangements. For each outcome, models were adjusted in the following manner. Model 1 was adjusted for age and sex. Model 2 was additionally adjusted for occupation, occupational exposure risk of SARS‐CoV‐2, history of vaccination, history of infection, infection prevention practice score, frequency of spending ≥ 30 min in the 3Cs (crowded places, close‐contact settings, and confined and enclosed spaces) without a mask, and frequency of having dinner in a group of ≥ 5 people for > 1 h. Histories of COVID‐19 vaccination and SARS‐CoV‐2 infection were adjusted in the analysis of SARS‐CoV‐2 incidence, while histories of influenza vaccination and infection within 1 year were adjusted in the analysis of influenza incidence. In the analyses for absenteeism due to own/family‐related events, histories of vaccination and infection for both illnesses were adjusted.

Statistical analyses were performed using Stata 18.0 (StataCorp LLC), and graphics were generated using GraphPad Prism 9 (GraphPad, Inc.). All *p* values were two‐sided, and *p* < 0.05 was considered statistically significant.

## 3 Results

### 3.1 Baseline Characteristics

Of the 1473 participants, 72% were female, and the median age was 40 years (Table 1). Participants who lived alone were younger, tended to be engaged in work at high risk of SARS‐CoV‐2 exposure, and more frequently performed infection‐risk behaviors in their leisure time compared with those with cohabitants. Among participants with cohabitants, those who lived with younger school‐aged children were younger and tended to have histories of SARS‐CoV‐2 infection and influenza infection compared with those who lived without school‐aged children.

**TABLE 1:** Baseline characteristics according to living arrangement.

	Total	Living arrangement			
		Living alone	Living with family, not including school‐aged children	Living with older school‐aged children	Living with younger school‐aged children
Participants	*N* = 1473	*N* = 556	*N* = 462	*N* = 213	*N* = 242
Age, year	40 (29–50)	30 (25–43)	45 (31–58)	49 (45–54)	40 (36–44)
Female, %	72	79	72	63	66
Job, %					
Doctor	14	14	10	19	19
Nurse	35	47	27	23	31
Allied healthcare worker	15	13	19	8	19
Researcher	16	10	17	21	23
Administrative staff	17	13	23	23	7
Others	3	3	4	5	2
Occupational exposure risk of SARS‐CoV‐2, %^a^					
Low	65	59	72	69	64
Moderate	19	22	15	18	20
High	16	19	13	14	16
COVID‐19‐related factors					
Vaccination, %					
0–2 dose	4	4	3	5	3
3–4 dose	46	47	44	39	52
5–6 dose	39	39	38	44	38
7–8 dose	12	10	15	12	7
Days since the last vaccination, day	351 (60–467)	352 (151–468)	350 (50–467)	346 (13–461)	354 (59–477)
Previous infection, %	72	70	66	75	83
Days since the last diagnosis, d	350 (130–483)	347 (133–449)	207 (122–448)	360 (139–496)	447 (187–588)
Influenza related factors					
Vaccination within 1 year, %	88	86	89	84	93
Days since the last vaccination, day	30 (27–35)	30 (22–35)	30 (27–34)	30 (28–34)	30 (27–35)
Previous infection within 1 year, %	6	4	2	7	17
Days since the last diagnosis, day	94 (36–369)	98 (38–370)	94 (29–158)	96 (49–127)	67 (35.5–370)
Infection prevention practice score^b^	6 (5–8)	6 (5–7)	6 (5–8)	6 (5–8)	6 (5–8)
Spending ≥ 30 min in the 3Cs without mask, %					
None	36	29	38	46	41
1–5 times	41	42	43	38	40
≥ 6 times	22	29	19	16	19
Having dinner in a group of ≥ 5 people for > 1 h, %					
None	45	44	47	49	42
1–5 times	48	48	47	48	53
≥ 6 times	7	9	6	3	5
No. of household members	2 (1–3)	1	2 (2–3)	4 (3–4)	4 (3–4)

*Note:* Data are presented as median (interquartile range) for continuous variables and percentage for categorical variables.

Abbreviations: 3Cs, crowded places, close‐contact settings, and confined and enclosed spaces; COVID‐19, coronavirus disease 2019; SARS‐CoV‐2, severe acute respiratory syndrome coronavirus 2.

^a^ Occupational SARS‐CoV‐2 exposure risk was categorized as low (those not engaged in COVID‐19–related work), moderate (those engaged in COVID‐19–related work without heavy exposure to SARS‐CoV‐2), or high (those heavily exposed to SARS‐CoV‐2).

^b^ Infection prevention practice score was calculated on the basis of the total score of adherences to avoiding the 3Cs, hand washing, wearing a mask, social distancing, and not touching the face, nose, or mouth, assigning 2 points to “always,” 1 point to “often,” and 0 points to others (“seldom” and “not at all”).

### 3.2 Absenteeism Due to COVID‐19 and Influenza Events

**FIGURE 1:**
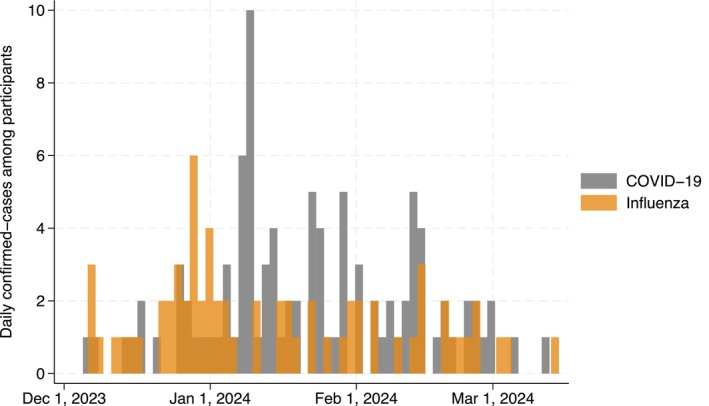
Daily confirmed‐cases of COVID‐19 and influenza during the follow‐up.

During the follow‐up (December 2023 to March 2024), we identified 98 and 66 participants with SARS‐CoV‐2 and influenza infections, respectively, with an incident rate of 7.4 and 5.0 per 10,000 person‐days, respectively (Figure 1). Four participants were infected with SARS‐CoV‐2 and influenza during the follow‐up. Of those, one was co‐infected with them simultaneously.

Among participants with cohabitants, 89 (9.7%) experienced absenteeism due to care for family members in relation to COVID‐19 or influenza events. Including absenteeism for infection, 164 (17.9%) participants with cohabitants experienced absenteeism due to own‐ or family‐related COVID‐19 or influenza events.

### 3.3 Association Between Living Arrangements and the Risk of Absenteeism

Compared with participants living with families without school‐aged children, those living with older school‐aged children had higher, but not statistically significant, risks of SARS‐CoV‐2 and influenza infections, with the HRs (95% CIs) of 1.51 (0.76–2.98) and 2.07 (0.88–4.85), respectively (Table 2). The HRs (95% CIs) for those living with younger school‐aged children were even higher, at 1.90 (0.97–3.69) for SARS‐CoV‐2 and 2.42 (1.04–5.66) for influenza. There were no substantial differences in the risks of SARS‐CoV‐2 and influenza infections between those living alone and those living with families without school‐aged children.

**TABLE 2:** Living with school‐aged children and the risk of absence among the staff of a tertiary hospital in Tokyo during the Twindemic of COVID‐19 and influenza (December 2023 to March 2024).

	Living arrangement			
	Living alone	Living with family, not including school‐aged children	Living with older school‐aged children	Living with younger school‐aged children
Reason for the absenteeism	*n* = 556	*n* = 462	*n* = 213	*n* = 242
Own SARS‐CoV‐2 infection				
Incidences/person‐days (%^a^)	37/49486 (7.5)	28/41409 (6.8)	14/19135 (7.3)	19/21709 (8.8)
Model 1^b^	0.98 (0.58–1.64)	Reference	1.17 (0.59–2.30)	1.37 (0.72–2.60)
Model 2^b^	0.92 (0.55–1.55)	Reference	1.51 (0.76–2.98)	1.90 (0.97–3.69)
Own influenza infection				
Incidences/person‐days (%^a^)	25/50056 (5.0)	15/41871 (3.6)	11/18938 (5.8)	15/21755 (6.9)
Model 1^b^	0.91 (0.47–1.77)	Reference	2.30 (0.99–5.35)	2.47 (1.08–5.64)
Model 2^b^	0.81 (0.41–1.59)	Reference	2.07 (0.88–4.85)	2.42 (1.04–5.66)
Family‐related events				
Incidences/person‐days (%^a^)	—	6/42695 (1.4)	24/19643 (12.2)	59/22458 (26.3)
Model 1^c^	—	Reference	5.79 (2.46–13.6)	11.4 (4.91–26.3)
Model 2^c^	—	Reference	6.25 (2.75–14.2)	10.9 (4.88–24.5)
Own/family‐related events				
Incidences/person‐days (%^a^)	—	44/42695 (10.3)	46/19643 (23.4)	74/22458 (33.0)
Model 1^c^	—	Reference	2.36 (1.58–3.52)	3.34 (2.33–4.79)
Model 2^c^	—	Reference	2.55 (1.72–3.77)	3.76 (2.59–5.46)

*Note:* Model 1 was adjusted for sex and age. Model 2 was adjusted for sex, age, occupation, occupational exposure risk of SARS‐CoV‐2, history of vaccination, history of infection, infection prevention practice score, spending ≥ 30 min in the 3Cs without mask, and having dinner in a group of ≥ 5 people for > 1 h. In the analyses for SARS‐CoV‐2 infection, histories of COVID‐19 vaccination and SARS‐CoV‐2 infection were adjusted. In the analyses for influenza infection, histories of influenza vaccination and infection were adjusted. In the analyses for family‐related and own/family‐related events, histories of vaccination and infection for both illnesses were adjusted.

Abbreviations: 3Cs, crowded places, close‐contact settings, and confined and enclosed spaces; COVID‐19, coronavirus disease 2019; SARS‐CoV‐2, severe acute respiratory syndrome coronavirus 2.

^a^ Incident rate per 10,000 person‐days.

^b^ Data are shown as a hazard ratio (95% CI) estimated by a Cox proportional hazard regression model.

^c^ Data are shown as an incident rate ratio (95% CI) estimated by a Poisson regression model with a robust variance estimator.

Compared with those living with cohabitants who were not school‐aged children, those living with older and younger school‐aged children had a significantly higher risk of absenteeism due to family care for COVID‐19 or influenza events, with the IRRs (95% CIs) of 6.25 (2.75–14.2) and 10.9 (4.88–24.5), respectively (Table 2). These groups also showed an increased risk of absenteeism due to any reason (own infection or family‐related COVID‐19 or Flu events), with IRRs (95% CIs) of 2.55 (1.72–3.77) and 3.76 (2.59–5.46), respectively.

### 3.4 Reasons for Absenteeism

**FIGURE 2:**
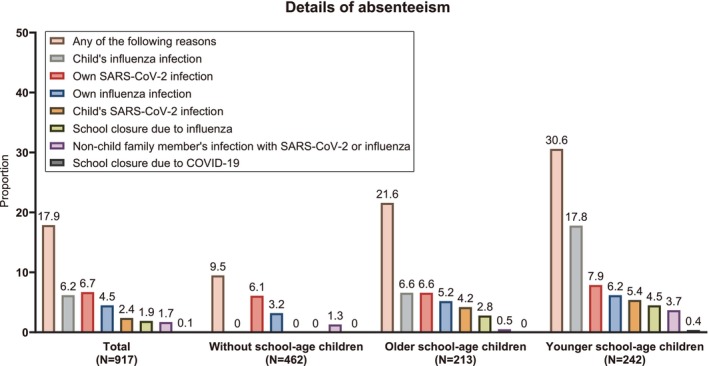
Reasons for absenteeism in relation to COVID‐19 or influenza events from December 2023 to March 2024 among participants with cohabitants (*N* = 917).

During the follow‐up, 17.9% of participants with cohabitants experienced absenteeism due to COVID‐19 or influenza‐related events (Figure 2). The main reasons were own infection with SARS‐CoV‐2 (6.7%) and child infection with influenza (6.2%), followed by own infection with influenza (4.5%), child infection with SARS‐CoV‐2 (2.4%), school closures due to influenza (1.9%), non‐child family infection with SARS‐CoV‐2 or influenza (1.7%), and school closures due to COVID‐19 (0.1%).

For participants with younger school‐aged children, 30.6% experienced absenteeism, predominantly due to child infection with influenza (17.8%), far surpassing the other reasons (0.4%–7.9%) (Figure 2). Among those with older school‐aged children, 21.6% experienced absenteeism, primarily due to child infection with influenza (6.6%) and own infection with SARS‐CoV‐2 (6.6%). For those without school‐aged children, 9.5% experienced absenteeism and the reasons were own infection with SARS‐CoV‐2 (6.1%) or influenza (3.2%) and non‐child family infection with SARS‐CoV‐2 or influenza (1.3%).

## 4 Discussion

During the Twindemic of COVID‐19 and influenza in the winter of 2023–2024 in Japan, at a tertiary hospital in Tokyo, HCWs living with school‐aged children had a higher risk of absenteeism due to COVID‐19 or influenza‐related events compared with those without school‐aged children. This risk was especially pronounced for HCWs with younger school‐aged children compared with those with older ones. This is the first study to show the impact of the Twindemic on the risk of absenteeism among HCWs.

In the study conducted in the pre‐Omicron phases, living with children was not identified as a risk factor for SARS‐CoV‐2 infection in adults [13]. Since the emergence of Omicron variants, children have become more susceptible to SARS‐CoV‐2 infection, increasing household transmission from children to adults [14]. In our previous study conducted during the Omicron BA.1/2 predominant wave (December 2021 to March 2022), HCWs living with younger school‐aged children had a 3.3‐hold higher risk of SARS‐CoV‐2 infection than those without school‐aged children [9]. The present study in the Omicron JN.1 wave (December 2023 to March 2024) also showed that HCWs with younger school‐aged children had a high risk of SARS‐CoV‐2 infection, albeit the relative risk decreased from 3.3 to 1.9. This risk attenuation could be influenced by the fact that COVID‐19 was less prevalent among younger school‐aged children during the present study (5.4%) compared with the previous study (11%) [9]. Nonetheless, it should be noted that the risk of SARS‐CoV‐2 infection was still high in HCWs with school‐aged children during the Omicron JN.1 wave.

Before the COVID‐19 pandemic, living with children is an established risk factor for contracting influenza in adults [15]. The present finding confirms this evidence and extends it to the era of the co‐circulation of COVID‐19 and influenza. In addition, we found that the association between living with younger school‐aged children and the risk of influenza infection was stronger than that for SARS‐CoV‐2 infection (IRR: 2.42 vs. 1.90). During the follow‐up periods (December 2023 to March 2024), influenza circulation was higher than COVID‐19 in Japan (Figure S1). In our findings, infections among children and school closures caused by influenza were more frequent than those by COVID‐19. These findings suggest that influenza circulated more widely than COVID‐19 among children during the 2023/2024 season in Japan, and this difference among children may have contributed to the observed disparity in infection risk among HCWs with or without younger school‐aged children.

In the present study, 24% of HCWs with younger school‐aged children experienced absences due to family care related to COVID‐19 or influenza events during the Twindemic. This proportion is notably lower than the 48% reported in our previous study on the COVID‐19 pandemic in 2022 [9]. The decrease in the rate of childcare‐related absenteeism from 2022 to 2024 could be explained by the relaxation of infection control standards following the revision of the Japanese Infectious Diseases Control Law in May 2023. Before May 2023, if children had close contact with COVID‐19 patients, they had to quarantine, necessitating their parents to leave work for childcare. This quarantine rule was lifted after May 2023. In addition, many Japanese municipalities have relaxed the standards for deciding school closures after May 2023. In our cohort, the proportion of absenteeism due to school closures significantly decreased from the previous [9] to present studies (34% to 4.9%). Our result suggests the possibility that the frequency of absenteeism due to childcare during the circulating acute respiratory infections could be associated more with the infection control policy in children than with the number of circulating viruses.

Some limitations should be acknowledged. Residual confounding factors may exist. We did not collect information on the histories of vaccination and infections for COVID‐19 and influenza among children, which could influence the children's susceptibility to contracting both illnesses and transmissibility from children to parents. In addition, our study was conducted in Japan and in the predominance wave of SARS‐CoV‐2 Omicron JN.1 and several seasonal influenza viruses (A/H1N1, A/H3N2, and B/Victoria); thus, caution should be exercised to generalize the Twindemic impact on the HCW's absenteeism to other nations with different policies to countermeasures against acute respiratory infections and to the situation of co‐circulating its other strains or other viruses.

## 5 Conclusion

During the Twindemic of COVID‐19 and influenza in the winter of 2023–2024 in Japan, HCWs with school‐aged children faced a high risk of contracting both illnesses and increased absenteeism due to caring for sick children and dealing with school closures. These findings emphasize the need for practical solutions to help HCWs balance their work and home responsibilities during such challenging times.

## Author Contributions

**Shohei Yamamoto:** conceptualization, writing – original draft, formal analysis, data curation, methodology, software, investigation, funding acquisition, visualization, project administration. **Tetsuya Mizoue:** conceptualization, writing – review and editing, supervision, methodology, funding acquisition, project administration, investigation, validation. **Maki Konishi:** data curation, software, investigation, writing – review and editing. **Kumi Horii:** data curation, investigation, resources, writing – review and editing. **Wataru Sugiura:** supervision, resources, project administration, writing – review and editing. **Norio Ohmagari:** writing – review and editing, supervision, resources, project administration.

## Conflicts of Interest

The authors declare no conflicts of interest.

### Peer Review

The peer review history for this article is available at https://www.webofscience.com/api/gateway/wos/peer‐review/10.1111/irv.70100.

## Supporting information


**Figure S1** Weekly cases per sentinel in Japan from May 2023 to April 2024

## Data Availability

The data that support the findings of this study are available on request from the corresponding author. The data are not publicly available due to privacy or ethical restrictions.
