# Supplementary material for: Living With School‐Aged Children and the Risk of Absenteeism Among Healthcare Workers During the Twindemic of COVID‐19 and Influenza
Source: Influenza Other Respir Viruses. 2025 Apr 21;19(4):e70100. doi: 10.1111/irv.70100 (PMC12010135; doi:10.1111/irv.70100)
Supplement: Supplementary file 1 — Figure S1 Weekly cases per sentinel in Japan from May 2023 to April 2024 [file IRV-19-e70100-s001.docx]

**Supplemental Appendix**

Living with school-aged children and the risk of absenteeism among healthcare workers

during the Twindemic of COVID-19 and Influenza

Shohei Yamamoto, Tetsuya Mizoue, Maki Konishi, Kumi Horii, Wataru Sugiura, Norio Ohmagari

[**Figure S1.** Weekly cases per sentinel in Japan from May 2023 to April 2024 2](#_Toc174622705)

**
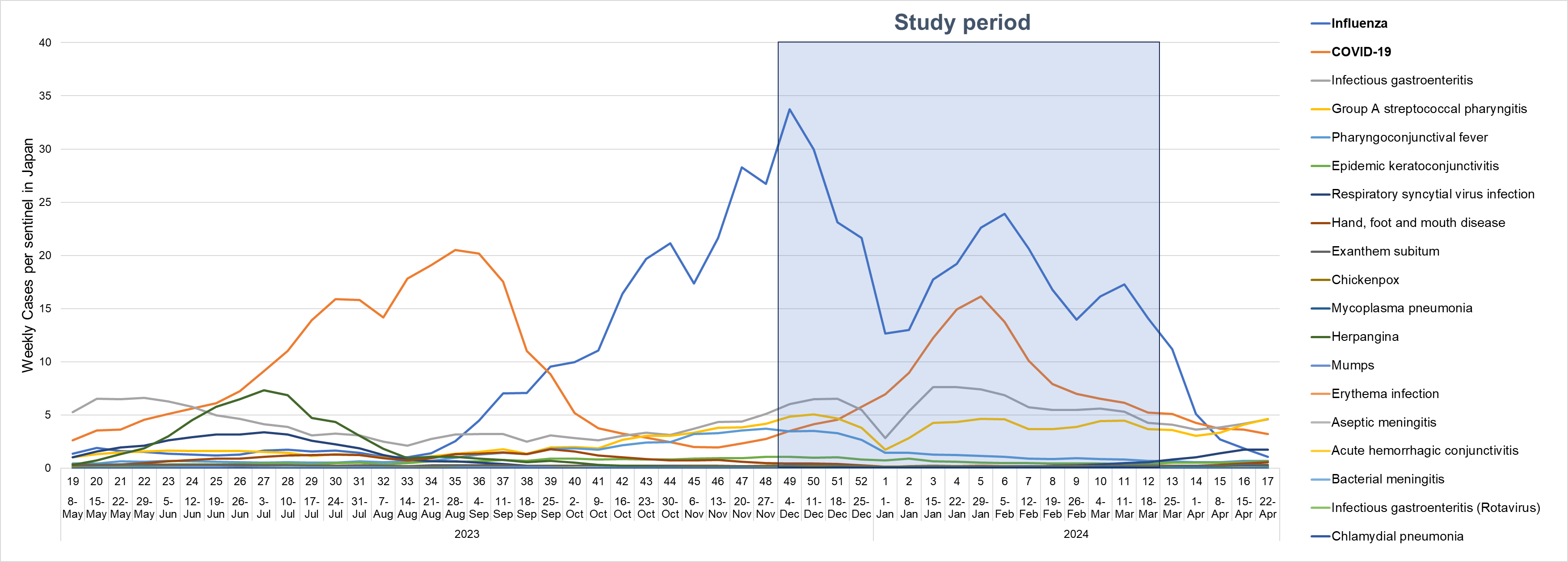
**

# **Figure S1.** Weekly cases per sentinel in Japan from May 2023 to April 2024
